# Effectiveness of Different Toothbrushing Methods on Plaque Accumulation and Gingival Health in Patients With Fixed Orthodontic Appliances

**DOI:** 10.7759/cureus.113862

**Published:** 2026-08-03

**Authors:** Merry Goyal, Sanjay Mittal, Baljeet Singh, Isha Aggarwal, Kanish Aggarwal, Pallavi Vishavkarma

**Affiliations:** 1 Department of Orthodontics and Dentofacial Orthopedics, Bhojia Dental College and Hospital, Baddi, IND; 2 Department of Periodontics, Himachal Dental College, Sunder Nagar, IND; 3 Department of Orthodontics and Dentofacial Orthopedics, T-Rex Dental Clinic, Nalagarh, IND

**Keywords:** dental plaque, gingivitis, oral hygiene, orthodontic appliances, tooth brushing

## Abstract

Introduction: Maintaining adequate oral hygiene during fixed orthodontic treatment is challenging because brackets, archwires, and ligatures promote plaque retention and increase the risk of gingival inflammation. Various toothbrush designs have been introduced to improve plaque removal; however, their comparative effectiveness in orthodontic patients remains unclear. We aimed to compare the effectiveness of different toothbrushing methods in maintaining plaque control and gingival health among patients undergoing fixed orthodontic treatment. Materials and methods: This prospective study included 90 participants undergoing fixed orthodontic treatment who were divided into five equal groups (n = 18 each) according to the prescribed toothbrushing method: conventional manual toothbrush, orthodontic manual toothbrush, powered electric toothbrush, conventional manual toothbrush with an interdental brush, and orthodontic manual toothbrush with an interdental brush. Plaque accumulation and gingival health were assessed at baseline and monthly intervals for six months using orthodontic plaque and gingival indices. Data were analyzed using two-way repeated-measures analysis of variance with appropriate post hoc tests, and statistical significance was established at p < 0.05. Results: All toothbrushing methods demonstrated progressive improvements in plaque and gingival scores during the six-month follow-up. Mean plaque index decreased from 1.16 ± 0.51 to 0.35 ± 0.12 in the conventional manual toothbrush group and from 1.07 ± 0.54 to 0.22 ± 0.12 in the powered electric toothbrush group. The gingival index decreased from 1.66 ± 0.48 to 0.39 ± 0.19 in the conventional manual toothbrush group and from 1.46 ± 0.61 to 0.28 ± 0.18 in the orthodontic manual toothbrush with interdental brush group. There was a significant main effect of time on both the plaque and gingival indices (p < 0.001). The group effect was significant for gingival index (p < 0.001) but not for plaque index (p = 0.178). No significant group × time interactions were observed. Post hoc analysis showed a significantly lower plaque index in the powered electric toothbrush group than in the conventional manual toothbrush group (p = 0.032). Conclusions: All evaluated toothbrushing methods improved oral hygiene and gingival health during fixed orthodontic treatment. The powered electric toothbrush showed the greatest plaque reduction and a statistically significant advantage over conventional manual toothbrushes in a post hoc comparison. In contrast, the orthodontic manual toothbrush combined with an interdental brush demonstrated the most favorable gingival health outcomes. Regular reinforcement of oral hygiene practices, together with appropriate toothbrush selection tailored to individual patient needs, may help minimize plaque-related complications during orthodontic treatment.

## Introduction

Maintaining optimal oral hygiene during fixed orthodontic treatment is challenging because brackets, bands, archwires, and ligatures create additional plaque-retentive areas that hinder effective tooth cleaning [1]. The increased accumulation of dental plaque around orthodontic appliances promotes the growth of pathogenic microorganisms, resulting in gingival inflammation, enamel demineralization, white spot lesions, and periodontal tissue destruction in severe cases [2]. Therefore, effective plaque control is considered an essential component of successful orthodontic treatment, helping preserve periodontal health and prevent treatment-related complications.

Mechanical plaque removal through regular toothbrushing remains the primary means of maintaining oral hygiene in orthodontic patients. However, conventional manual toothbrushes often fail to adequately clean areas adjacent to brackets and beneath archwires owing to limited access [3]. To overcome these challenges, several oral hygiene aids have been developed, including orthodontic-specific manual toothbrushes with V-shaped bristle configurations. These include orthodontic-specific manual toothbrushes with V-shaped bristle configurations, powered electric toothbrushes that provide automated oscillatory movements, and interdental brushes designed to clean interdental and subarchwire regions [4,5]. Although these devices are widely recommended, their relative effectiveness in improving plaque control and gingival health remains debatable. Irrespective of the toothbrush used, regular periodontal evaluation, reinforcement of oral hygiene instructions, and periodic professional prophylaxis remain essential adjuncts for maintaining periodontal health during fixed orthodontic treatment.

Previous studies have reported varying outcomes when comparing the different toothbrushing methods in orthodontic patients. While some investigations have demonstrated superior plaque removal with powered electric toothbrushes, others have found comparable clinical outcomes between powered and manual toothbrushes when patients receive appropriate oral hygiene instructions and regular motivation [6-8]. Likewise, the additional benefit of interdental brushes alongside routine toothbrushing has not been consistently established. These inconsistencies may be attributed to differences in the study design, follow-up duration, patient compliance, brushing techniques, and outcome measures. Consequently, further comparative clinical studies are required to identify practical and effective oral hygiene methods for individuals undergoing fixed orthodontic treatments. The present study aimed to compare the effectiveness of five toothbrushing methods in maintaining oral hygiene among patients undergoing fixed orthodontic treatment over a six-month follow-up period. The primary objective was to compare the effectiveness of these toothbrushing methods in reducing plaque accumulation, as assessed by the Plaque Index. The secondary objective was to compare their effectiveness in improving gingival health, as assessed by the gingival index, over the same follow-up period.

## Materials and methods

Study design and setting

This prospective comparative clinical study was conducted in the Department of Orthodontics and Dentofacial Orthopedics, Bhojia Dental College and Hospital, Baddi, Himachal Pradesh, India, between December 2015 and December 2016, after obtaining approval from the Institutional Ethical Committee of Bhojia Dental College and Hospital (approval number: BDCH/BUDH/IEC/152, dated October 17, 2015). Written informed consent was obtained from all participants, their parents, or guardians before enrollment. This study was conducted in accordance with the ethical principles of the Declaration of Helsinki.

Sample size estimation

The sample size was estimated using the G*Power software version 3.1.9.7 (Heinrich Heine University Düsseldorf, Düsseldorf, Germany). Based on a previous study evaluating plaque index among different toothbrushing methods, an effect size (f) of 0.40 was considered [9]. Assuming a statistical power of 80%, a two-sided significance level of 5%, and comparison among five independent groups using the F-test (one-way analysis of variance, ANOVA), the minimum required sample size was calculated as 90 participants. Accordingly, 90 patients were included in the study, with 18 participants assigned to each group.

Participant selection

Patients aged 12-22 years undergoing treatment with fixed pre-adjusted edgewise appliances in both arches were screened for eligibility. Individuals who had been undergoing orthodontic treatment for not more than six months, were systemically healthy, had permanent dentition, and were willing to comply with the study protocol were included. Patients with systemic diseases affecting periodontal health, current steroid or hormonal therapy, compromised manual dexterity, active periodontal disease, calculus deposits, untreated dental caries, professional oral prophylaxis within the previous three months, systemic antibiotic therapy within one month prior to enrollment, or use of antimicrobial mouth rinses during the study period were excluded from the study.

Group allocation

Ninety eligible participants were consecutively assigned to five groups (n = 18 each) according to the prescribed toothbrushing method in this prospective comparative clinical study. Group A used a conventional manual toothbrush (Colgate SlimSoft Tri-Tip, soft bristles; Colgate-Palmolive Company, NY, USA), Group B used an orthodontic manual toothbrush (Thermoseal Ortho; ICPA Health Products Ltd., Gujarat, India), Group C used a powered electric toothbrush (Oral-B CrossAction Power Dual Clean; Procter & Gamble, Cincinnati, OH, USA), Group D used a conventional manual toothbrush in combination with an interdental brush (Thermoseal Proxa NS; ICPA Health Products Ltd., Gujarat, India), and Group E used an orthodontic manual toothbrush in combination with an interdental brush. Allocation was non-randomized and based on sequential enrollment of eligible patients to ensure balanced group sizes while reflecting real-world prescription practices. Participants continued using their assigned toothbrushing method throughout the six-month follow-up period. Owing to the obvious differences among the brushing devices, blinding of participants and the examiner was not feasible. However, all clinical assessments were performed by a single calibrated examiner using standardized assessment protocols to minimize measurement variability.

Oral hygiene instructions

At baseline, all participants received standardized oral hygiene instructions from the same trained investigator. Participants in Group A (conventional manual toothbrush) were instructed to use the modified Bass technique, in which the bristles were positioned at approximately 45° to the gingival margin and gentle vibratory strokes were applied, followed by a rolling motion toward the occlusal surface [10]. Participants in Groups B (orthodontic manual toothbrush) and E (orthodontic manual toothbrush with interdental brush) were instructed to use the orthodontic brushing technique, positioning the V-shaped bristles simultaneously above and below the orthodontic brackets at approximately 45° to the tooth surface. Gentle back-and-forth strokes were used to clean the gingival, bracket, and occlusal surfaces, ensuring adequate cleaning around brackets and beneath the archwire. Participants in Group C (powered electric toothbrush) were instructed to use the toothbrush according to the manufacturer's recommendations. The brush head was placed sequentially on the buccal, lingual, and occlusal surfaces of each tooth, allowing the oscillating-rotating action of the brush to perform the cleaning without additional manual scrubbing. The brush head was held against each tooth for approximately two to three seconds before moving to the next tooth. Participants in Groups D and E were instructed to use an interdental brush (Thermoseal Proxa NS, 0.7 mm; ICPA Health Products Ltd., Gujarat, India) to clean beneath the archwire and in the interproximal areas. All participants were advised to brush twice daily, after breakfast and before bedtime, for a minimum duration of two minutes throughout the six-month study period.

Clinical assessment

Clinical examinations were performed at baseline (T0) and at monthly intervals (T1-T6) over a six-month follow-up period by a single calibrated examiner. Plaque accumulation was assessed using the orthodontic plaque index [11], which scores plaque on the mesial, distal, gingival, and mid-bracket surfaces of each tooth. Gingival health was evaluated using the Löe and Silness gingival index [12] with a Williams periodontal probe. Plaque assessment was performed before the gingival examination at every visit to minimize bias from probing-induced bleeding.

Examiner calibration

To ensure measurement reliability, examiner calibration was performed prior to study commencement. A subset of participants was re-evaluated by the examiner on two separate occasions, one week apart. Intra-examiner reliability was assessed using the intraclass correlation coefficient (ICC), yielding excellent agreement for the orthodontic plaque index (ICC = 0.986) and gingival index (ICC = 0.956).

Statistical analysis

Data were analyzed using statistical software (IBM Corp. Released 2011. IBM SPSS Statistics for Windows, Version 20.0. Armonk, NY). Normality of the data distribution was assessed using the Shapiro-Wilk test. Continuous variables are presented as the mean ± standard deviation (SD). Changes in plaque and gingival indices over time and among the five toothbrushing method groups were analyzed using two-way repeated-measures ANOVA. When significant differences were identified, intergroup comparisons were performed using Tukey's honestly significant difference (HSD) post hoc test. In contrast, intragroup comparisons across follow-up visits were conducted using Bonferroni-adjusted post hoc analysis. Statistical significance was set at p < 0.05.

## Results

Ninety participants were included in the study, with 18 (20.0%) participants in each of the five toothbrushing method groups. The study population comprised 31 males (34.4%) and 59 females (65.6%). The baseline demographic characteristics, including age, sex distribution, plaque index, and gingival index, were comparable among the groups, with no statistically significant differences (all p > 0.05), indicating baseline homogeneity (Table 1).

**Table 1:** Baseline demographic and clinical characteristics of participants according to the toothbrushing method groups Data are presented as mean ± SD or number (%), and one-way ANOVA was used for continuous variables; a chi-square test was used for categorical variables; statistical significance was considered at p < 0.05. CMT: conventional manual toothbrush, OMT: orthodontic manual toothbrush, PET: powered electric toothbrush, CMT + IB: conventional manual toothbrush with interdental brush, OMT + IB: orthodontic manual toothbrush with interdental brush, SD: standard deviation, ANOVA: analysis of variance

Parameter		A = CMT (n = 18)	B = OMT (n = 18)	C = PET (n = 18)	D = CMT + IB (n = 18)	E = OMT + IB (n = 18)	Test value	p-value	Effect size
Age (years)	Mean ± SD	17.80 ± 2.23	17.50 ± 2.15	17.23 ± 2.67	16.44 ± 1.80	17.42 ± 2.10	1.18	0.326	0.054
Sex, n (%)	Male	5 (27.7)	6 (33.3)	8 (44.4)	5 (27.7)	7 (38.9)	1.44	0.837	0.126
	Female	13 (72.3)	12 (66.7)	10 (55.6)	13 (72.3)	11 (61.1)			
Orthodontic plaque index	Mean ± SD	1.16 ± 0.51	1.11 ± 0.58	1.07 ± 0.54	1.24 ± 0.67	1.23 ± 0.60	0.28	0.891	0.013
Gingival index	Mean ± SD	1.66 ± 0.48	1.14 ± 0.55	1.11 ± 0.56	1.27 ± 0.69	1.46 ± 0.61	3.16	0.118	0.130

Both orthodontic plaque index and gingival index showed a progressive reduction in all five groups throughout the six-month follow-up period (Table 2). The powered electric toothbrush group demonstrated the lowest orthodontic plaque index at the six-month follow-up. In contrast, the orthodontic manual toothbrush combined with an interdental brush showed the lowest gingival index at the final evaluation.

**Table 2:** Longitudinal changes in orthodontic plaque index and gingival index among the five toothbrushing method groups during the six-month follow-up Data are presented as mean ± SD; measurements were recorded at baseline (T0) and monthly follow-up visits (T1-T6), and descriptive statistics are presented for each study group. T0 = baseline, T1 = month 1, T2 = month 2, T3 = month 3, T4 = month 4, T5 = month 5, and T6 = month 6. CMT: conventional manual toothbrush, OMT: orthodontic manual toothbrush, PET: powered electric toothbrush, CMT + IB: conventional manual toothbrush with interdental brush, OMT + IB: orthodontic manual toothbrush with interdental brush, SD: standard deviation

Parameter	Group	Baseline (T0)	Month 1 (T1)	Month 2 (T2)	Month 3 (T3)	Month 4 (T4)	Month 5 (T5)	Month 6 (T6)
Orthodontic plaque index	CMT	1.16 ± 0.51	0.77 ± 0.34	0.63 ± 0.28	0.54 ± 0.27	0.51 ± 0.33	0.43 ± 0.18	0.35 ± 0.12
	OMT	1.11 ± 0.58	0.64 ± 0.46	0.54 ± 0.29	0.52 ± 0.25	0.45 ± 0.24	0.37 ± 0.19	0.30 ± 0.10
	PET	1.07 ± 0.54	0.66 ± 0.43	0.53 ± 0.28	0.57 ± 0.29	0.40 ± 0.20	0.30 ± 0.15	0.22 ± 0.12
	CMT+IB	1.24 ± 0.67	0.82 ± 0.53	0.54 ± 0.31	0.52 ± 0.28	0.47 ± 0.25	0.39 ± 0.12	0.32 ± 0.12
	OMT+IB	1.23 ± 0.60	0.77 ± 0.43	0.60 ± 0.39	0.45 ± 0.17	0.39 ± 0.15	0.44 ± 0.26	0.31 ± 0.14
Gingival index	CMT	1.66 ± 0.48	0.97 ± 0.52	0.74 ± 0.49	0.67 ± 0.40	0.57 ± 0.48	0.40 ± 0.20	0.39 ± 0.19
	OMT	1.14 ± 0.55	0.63 ± 0.44	0.54 ± 0.29	0.52 ± 0.25	0.46 ± 0.24	0.37 ± 0.19	0.30 ± 0.11
	PET	1.11 ± 0.56	0.61 ± 0.31	0.61 ± 0.32	0.42 ± 0.16	0.37 ± 0.24	0.34 ± 0.22	0.30 ± 0.18
	CMT+IB	1.27 ± 0.69	0.91 ± 0.56	0.68 ± 0.40	0.52 ± 0.25	0.51 ± 0.27	0.45 ± 0.24	0.34 ± 0.12
	OMT+IB	1.46 ± 0.61	0.91 ± 0.50	0.56 ± 0.29	0.50 ± 0.31	0.33 ± 0.15	0.39 ± 0.26	0.28 ± 0.18

Two-way repeated-measures ANOVA demonstrated a significant effect of time on both orthodontic plaque index and gingival index (both p < 0.001). The main effect of group was significant for gingival index (p < 0.001) but not for plaque index (p = 0.178). No significant group × time interactions were observed for either outcome (Table 3).

**Table 3:** Two-way repeated-measures analysis of variance for orthodontic plaque index and gingival index during the study period Data are presented as sum of squares, degrees of freedom, mean square, F-value, p-value, and partial eta squared (η²p). Two-way repeated-measures ANOVA was used to evaluate the effects of group, time, and group × time interaction. *p < 0.05 indicates statistical significance. ANOVA: analysis of variance, SS: sum of squares, MS: mean square

Parameters	Source of variation	SS	df	MS	F-value	p-value	Effect size (η²p)
Orthodontic plaque index	Group (A vs B vs C vs D vs E)	0.73	4	0.181	1.58	0.178	0.010
	Time (T0 to T6)	45.00	6	7.495	65.13	<0.001*	0.396
	Group × Time (Interaction)	0.96	24	0.039	0.347	0.997	0.013
Gingival index	Group (A vs B vs C vs D vs E)	4.30	4	1.074	7.98	<0.001*	0.050
	Time (T0 to T6)	64.30	6	10.715	79.56	<0.001*	0.445
	Group × Time (Interaction)	3.72	24	0.155	1.15	0.280	0.044

Intergroup post hoc analysis revealed that a significantly higher plaque index was observed only between group A and group C (mean difference = 0.091, p = 0.032). In contrast, all other pairwise comparisons were not statistically significant (p > 0.05). In contrast, the gingival index showed several significant intergroup differences. Group A exhibited significantly higher gingival index values compared with groups B, C, D, and E (all p ≤ 0.026). Additionally, significant differences were observed between groups B and D (p = 0.026), groups C and D (p = 0.005), and groups C and E (p = 0.038). No significant differences in gingival index were found between groups B and C, groups B and E, or groups D and E (p > 0.05) (Table 4).

**Table 4:** Intergroup pairwise comparisons of orthodontic plaque index and gingival index among the toothbrushing method groups Data are presented as mean differences, t-values, and p-values. Tukey's HSD post hoc test was used for pairwise intergroup comparisons; *p < 0.05 indicates statistical significance. A = conventional manual toothbrush, B = orthodontic manual toothbrush, C = powered electric toothbrush, D = conventional manual toothbrush with interdental brush, and E = orthodontic manual toothbrush with interdental brush. HSD: honestly significant difference

Comparison	Orthodontic plaque index			Gingival index		
	Mean difference	t-value	p-value	Mean difference	t-value	p-value
A vs B	0.065	1.539	0.124	0.205	4.452	<0.001*
A vs C	0.091	2.141	0.032*	0.234	5.071	<0.001*
A vs D	0.012	0.301	0.764	0.108	2.225	0.026*
A vs E	0.028	0.667	0.505	0.138	2.998	0.003*
B vs C	0.025	0.602	0.547	0.028	0.619	0.536
B vs D	–0.052	–1.239	0.216	–0.102	–2.227	0.026*
B vs E	–0.037	–0.871	0.384	–0.067	–1.455	0.146
C vs D	–0.078	–1.840	0.066	–0.131	–2.846	0.005*
C vs E	–0.062	–1.473	0.141	–0.095	–2.074	0.038*
D vs E	0.015	0.367	0.714	0.035	0.773	0.440

Within-group analysis demonstrated significant reductions in plaque index from baseline to subsequent follow-up visits in all five groups, confirming improvement in oral hygiene over the study period (Table 5).

**Table 5:** Intragroup pairwise comparison of orthodontic plaque index during the six-month follow-up Data are presented as t-values (p-values). A Bonferroni-adjusted post hoc test was used for multiple pairwise comparisons within each group. Bonferroni-corrected statistical significance was considered at p < 0.0024; *p < 0.0024 indicates statistical significance. T0 = baseline, T1 = month 1, T2 = month 2, T3 = month 3, T4 = month 4, T5 = month 5, and T6 = month 6. CMT: conventional manual toothbrush, OMT: orthodontic manual toothbrush, PET: powered electric toothbrush, CMT + IB: conventional manual toothbrush with interdental brush, OMT + IB: orthodontic manual toothbrush with interdental brush

Comparison	Orthodontic plaque index				
	CMT (n = 18)	OMT (n = 18)	PET (n = 18)	CMT + IB (n = 18)	OMT + IB (n = 18)
T0 vs T1	2.699 (0.011)	2.693 (0.011)	2.375 (0.031)	2.087 (0.044)	2.570 (0.021)
T0 vs T2	3.866 (<0.001*)	3.730 (<0.001*)	3.550 (0.003)	4.023 (<0.001*)	3.629 (0.002*)
T0 vs T3	4.559 (<0.001*)	3.962 (<0.001*)	3.264 (0.005)	4.208 (<0.001*)	5.155 (<0.001*)
T0 vs T4	4.538 (<0.001*)	4.460 (<0.001*)	4.653 (<0.001*)	4.567 (<0.001*)	5.600 (<0.001*)
T0 vs T5	5.726 (<0.001*)	5.146 (<0.001*)	5.496 (<0.001*)	5.299 (<0.001*)	4.981 (<0.001*)
T0 vs T6	6.559 (<0.001*)	5.840 (<0.001*)	6.147 (<0.001*)	5.736 (<0.001*)	6.158 (<0.001*)
T1 vs T2	1.349 (0.186)	0.780 (0.441)	1.013 (0.328)	1.935 (0.061)	1.207 (0.245)
T1 vs T3	2.248 (0.031)	0.972 (0.338)	0.694 (0.499)	2.123 (0.041)	2.852 (0.012)
T1 vs T4	2.327 (0.026)	1.554 (0.129)	2.192 (0.045)	2.535 (0.016)	3.439 (0.004)
T1 vs T5	3.749 (<0.001*)	2.302 (0.027)	3.163 (0.007)	3.357 (0.002*)	2.707 (0.016)
T1 vs T6	4.942 (<0.001*)	3.066 (0.004)	3.943 (<0.001*)	3.903 (<0.001*)	4.193 (<0.001*)
T2 vs T3	0.981 (0.333)	0.222 (0.826)	–0.397 (0.697)	0.203 (0.840)	1.454 (0.165)
T2 vs T4	1.176 (0.247)	1.014 (0.317)	1.512 (0.152)	0.746 (0.461)	2.073 (0.055)
T2 vs T5	2.549 (0.016)	2.081 (0.045)	2.897 (0.011)	1.916 (0.064)	1.407 (0.179)
T2 vs T6	3.899 (<0.001*)	3.320 (0.002*)	4.069 (0.001*)	2.810 (0.008)	2.886 (0.011)
T3 vs T4	0.298 (0.767)	0.857 (0.397)	1.930 (0.073)	0.565 (0.576)	1.091 (0.291)
T3 vs T5	1.438 (0.159)	2.027 (0.051)	3.309 (0.005)	1.811 (0.079)	0.133 (0.896)
T3 vs T6	2.728 (0.010)	3.465 (0.002*)	4.459 (<0.001*)	2.786 (0.009)	2.622 (0.019)
T4 vs T5	0.903 (0.373)	1.108 (0.276)	1.600 (0.131)	1.225 (0.229)	–0.687 (0.502)
T4 vs T6	1.932 (0.062)	2.447 (0.019)	3.088 (0.007)	2.297 (0.028)	1.607 (0.128)
T5 vs T6	1.569 (0.126)	1.383 (0.176)	1.663 (0.117)	1.750 (0.089)	1.816 (0.088)

Bonferroni-adjusted intragroup pairwise comparisons demonstrated significant reductions in gingival index over the six-month follow-up period in all toothbrushing method groups (Table 6). The most consistent significant changes occurred between baseline and the later follow-up periods, indicating sustained improvement in gingival health across all toothbrushing methods.

**Table 6:** Intragroup pairwise comparison of gingival index during the six-month follow-up Data are presented as t-values (p-values). A Bonferroni-adjusted post hoc test was used for multiple pairwise comparisons within each group. Bonferroni-corrected statistical significance was considered at p < 0.0024; *p < 0.0024 indicates statistical significance. T0 = baseline, T1 = month 1, T2 = month 2, T3 = month 3, T4 = month 4, T5 = month 5, and T6 = month 6. CMT: conventional manual toothbrush, OMT: orthodontic manual toothbrush, PET: powered electric toothbrush, CMT + IB: conventional manual toothbrush with interdental brush, OMT + IB: orthodontic manual toothbrush with interdental brush

Comparison	Gingival index				
	CMT (n = 18)	OMT (n = 18)	PET (n = 18)	CMT + IB (n = 18)	OMT + IB (n = 18)
T0 vs T1	4.14 (<0.001*)	3.07 (0.004)	3.13 (0.004)	1.72 (0.095)	2.88 (0.007)
T0 vs T2	5.69 (<0.001*)	4.09 (<0.001*)	3.10 (0.004)	3.14 (0.004)	5.50 (<0.001*)
T0 vs T3	6.72 (<0.001*)	4.35 (<0.001*)	4.74 (<0.001*)	4.34 (<0.001*)	5.78 (<0.001*)
T0 vs T4	6.81 (<0.001*)	4.81 (<0.001*)	4.86 (<0.001*)	4.35 (<0.001*)	7.42 (<0.001*)
T0 vs T5	10.28 (<0.001*)	5.62 (<0.001*)	5.12 (<0.001*)	4.77 (<0.001*)	6.65 (<0.001*)
T0 vs T6	10.43 (<0.001*)	6.35 (<0.001*)	5.51 (<0.001*)	5.64 (<0.001*)	7.65 (<0.001*)
T1 vs T2	1.37 (0.180)	0.72 (0.480)	0.00 (1.000)	1.42 (0.165)	2.50 (0.018)
T1 vs T3	1.94 (0.061)	0.92 (0.364)	2.18 (0.038)	2.70 (0.011)	2.87 (0.007)
T1 vs T4	2.40 (0.022)	1.44 (0.160)	2.45 (0.022)	2.73 (0.010)	4.58 (<0.001*)
T1 vs T5	4.35 (<0.001*)	2.30 (0.028)	2.84 (0.009)	3.20 (0.003)	3.80 (<0.001*)
T1 vs T6	4.45 (<0.001*)	3.08 (0.004)	3.47 (0.002*)	4.23 (< 0.001*)	4.89 (<0.001*)
T2 vs T3	0.47 (0.642)	0.22 (0.827)	2.12 (0.043)	1.44 (0.160)	0.58 (0.565)
T2 vs T4	1.05 (0.301)	0.90 (0.375)	2.40 (0.024)	1.49 (0.147)	2.92 (0.007)
T2 vs T5	2.73 (0.010)	2.08 (0.045)	2.79 (0.010)	2.09 (0.045)	1.80 (0.082)
T2 vs T6	2.83 (0.008)	3.27 (0.003)	3.38 (0.002*)	3.45 (0.002*)	3.39 (0.002*)
T3 vs T4	0.68 (0.501)	0.73 (0.470)	0.69 (0.495)	0.11 (0.913)	2.03 (0.052)
T3 vs T5	2.56 (0.015)	2.02 (0.051)	1.18 (0.248)	0.85 (0.402)	1.12 (0.271)
T3 vs T6	2.68 (0.011)	3.39 (0.002*)	2.00 (0.055)	2.74 (0.010)	2.52 (0.018)
T4 vs T5	1.39 (0.174)	1.25 (0.220)	0.37 (0.715)	0.70 (0.490)	-0.82 (0.421)
T4 vs T6	1.48 (0.149)	2.56 (0.015)	0.94 (0.355)	2.43 (0.021)	0.88 (0.388)
T5 vs T6	0.15 (0.882)	1.35 (0.187)	0.57 (0.574)	1.74 (0.092)	1.43 (0.163)

## Discussion

This prospective comparative study evaluated the effectiveness of five different toothbrushing methods for maintaining oral hygiene in patients undergoing fixed orthodontic treatment over a six-month period. The present findings demonstrate that all five toothbrushing methods produced progressive reductions in plaque accumulation and gingival inflammation during the follow-up period, indicating that regular oral hygiene reinforcement, irrespective of the toothbrushing method used, contributes substantially to improved periodontal health in orthodontic patients.

A significant reduction in the orthodontic plaque index was observed over time in all groups, with the powered electric toothbrush demonstrating the greatest reduction at the end of six months. Although the overall group effect for plaque index was not statistically significant, the powered electric toothbrush performed significantly better than the conventional manual toothbrush during the intergroup comparison. The superior performance of the powered electric toothbrush may be attributed to its oscillating-rotating motion, which produces uniform mechanical plaque disruption independent of the user's brushing technique [8]. This feature is particularly advantageous for orthodontic patients in whom brackets and archwires limit access to tooth surfaces and compromise the effectiveness of conventional brushing. Furthermore, powered electric toothbrushes require less manual dexterity and provide consistent cleaning efficiency, thereby improving plaque removal around the orthodontic attachments.

These observations are consistent with those reported by Wilcoxon et al. [13], Heintze et al. [14], and Erbe et al. [15], who demonstrated superior plaque control with powered electric toothbrushes compared to conventional manual toothbrushes in patients wearing fixed orthodontic appliances. Similarly, Sharma et al. [7] reported improved oral hygiene scores among orthodontic patients who used powered electric toothbrushes. By contrast, Clerehugh et al. [16] and Heasman et al. [17] observed no significant differences between powered and manual toothbrushes when participants received repeated oral hygiene instructions and remained highly motivated. Such differences among studies may be explained by variations in the type of powered electric toothbrush evaluated, participant compliance, brush head positioning, brushing duration, adherence to the manufacturer's instructions, follow-up duration, and study design, rather than differences in manual brushing technique.

Gingival health also improved significantly throughout the study, with the orthodontic manual toothbrush combined with an interdental brush demonstrating the lowest gingival index at the six-month evaluation. Although improvements occurred in all groups, significant intergroup differences suggested that the brush design may influence gingival health more than plaque removal alone. Orthodontic toothbrushes possess a V-shaped bristle arrangement that facilitates cleaning around the brackets while minimizing trauma to the gingival tissues. The additional use of an interdental brush further enhances cleaning beneath the archwire and within the embrasure areas that are difficult to access using conventional toothbrushes [18]. Consequently, the improved plaque disruption in these inaccessible regions likely contributed to the greater reduction in gingival inflammation observed in this group.

The present findings agree with those of a previous systematic review that reported improved plaque removal and gingival health with orthodontic toothbrushes compared with conventional manual toothbrushes [19]. The findings of the present study differ from those reported by Kossack et al. [20], who found that long-term improvement in plaque and gingival scores was primarily attributable to the adjunctive interdental cleaning aid rather than the type of toothbrush used. They also observed that patients with good baseline oral hygiene showed little additional benefit from the evaluated cleaning devices. The differences between the two studies may be explained by variations in study design, use of a sonic toothbrush with a powered interdental cleaning device, crossover protocol, baseline oral hygiene status of the participants, and differences in the clinical indices used to evaluate oral hygiene outcomes.

The significant effect of time emphasizes the importance of continuous oral hygiene motivation during orthodontic treatment. Regular reinforcement at follow-up appointments likely improved patient awareness and compliance, contributing to progressive reductions in plaque accumulation irrespective of the toothbrushing method. Similar findings have been reported by Marini et al. [21], who demonstrated that repeated oral hygiene motivation independently improves periodontal outcomes in orthodontic patients, regardless of the toothbrush used. Therefore, professional reinforcement is an integral component of orthodontic treatment protocols. The present study has several strengths, including its prospective longitudinal design, standardized oral hygiene instructions, comparison of five commonly used toothbrushing methods, use of validated plaque and gingival indices, and excellent examiner calibration, all of which enhance the reliability and clinical applicability of the findings.

Clinical recommendations

From a clinical perspective, although all evaluated toothbrushing methods improve oral hygiene when accompanied by regular instruction and reinforcement, powered electric toothbrushes may provide superior plaque control. In contrast, orthodontic toothbrushes combined with interdental brushes may offer additional benefits for maintaining gingival health. Clinicians should individualize toothbrush selection according to patient-specific factors such as age, manual dexterity, motivation level, treatment complexity, and affordability. Powered electric toothbrushes are particularly recommended for patients requiring maximal plaque reduction or those with limited manual dexterity. In contrast, the combination of an orthodontic manual toothbrush with an interdental brush is preferable when gingival inflammation control is the primary concern. Regardless of the method chosen, regular reinforcement of oral hygiene instructions and motivation at every follow-up appointment is essential to maximize compliance and achieve sustained improvements in periodontal health throughout fixed orthodontic treatment. Such an approach, combined with periodic professional prophylaxis and regular periodontal visits, can effectively minimize plaque-related complications, including gingivitis, gingival enlargement, and white spot lesions.

Limitations

The present study has several limitations that should be considered when interpreting the findings. The study evaluated clinical periodontal outcomes using plaque and gingival indices. Still, it did not assess microbiological parameters, enamel demineralization (white spot lesions), patient-reported outcomes, or objective measures of participant adherence. Additionally, the prospective non-randomized and non-blinded design may have introduced selection and measurement bias, although standardized assessment protocols and examiner calibration were employed to minimize these effects. Furthermore, the single-center setting and inclusion of participants aged 12-22 years may limit the generalizability of the findings to other populations. Therefore, the results should be interpreted with caution. Future multicenter randomized controlled trials incorporating objective adherence monitoring, microbiological and enamel health assessments, and patient-reported outcome measures are warranted to validate these findings.

## Conclusions

Within the limitations of this prospective comparative study, the use of orthodontic toothbrushes and adjunctive interdental brushes was associated with improved plaque control and gingival health compared with conventional toothbrushing methods in patients undergoing fixed orthodontic treatment. These findings suggest that these oral hygiene aids may provide clinical benefits when used as part of routine orthodontic care. However, the results should be interpreted with caution owing to the study design and the absence of objective adherence assessment. Further randomized controlled trials with blinded outcome assessment, objective adherence monitoring, and patient-reported outcome measures are warranted to confirm these findings and establish the comparative effectiveness of different oral hygiene devices.
